# The coevolution of toxin and antitoxin genes drives the dynamics of bacterial addiction complexes and intragenomic conflict

**DOI:** 10.1098/rspb.2012.0942

**Published:** 2012-07-11

**Authors:** Daniel J. Rankin, Leighton A. Turner, Jack A. Heinemann, Sam P. Brown

**Affiliations:** 1 Institute of Evolutionary Biology and Environmental Studies, University of Zürich, Building Y27, Winterthurerstrasse 190, 8057 Zürich, Switzerland; 2 Swiss Institute of Bioinformatics, Quartier Sorge Bâtiment Génopode, 1015 Lausanne, Switzerland; 3 School of Biological Sciences, University of Canterbury, Christchurch, New Zealand; 4 Department of Zoology, University of Oxford, South Parks Road, Oxford OX1 3PS, UK; 5 Centre for Immunity, Infection and Evolution, University of Edinburgh, West Mains Road, Edinburgh EH9 3JT, UK

**Keywords:** genomic conflict, plasmid evolution, horizontal gene transfer, post-segregational killing, genetic addiction, toxin–antitoxin systems

## Abstract

Bacterial genomes commonly contain ‘addiction’ gene complexes that code for both a toxin and a corresponding antitoxin. As long as both genes are expressed, cells carrying the complex can remain healthy. However, loss of the complex (including segregational loss in daughter cells) can entail death of the cell. We develop a theoretical model to explore a number of evolutionary puzzles posed by toxin–antitoxin (TA) population biology. We first extend earlier results demonstrating that TA complexes can spread on plasmids, as an adaptation to plasmid competition in spatially structured environments, and highlight the role of kin selection. We then considered the emergence of TA complexes on plasmids from previously unlinked toxin and antitoxin genes. We find that one of these traits must offer at least initially a direct advantage in some but not all environments encountered by the evolving plasmid population. Finally, our study predicts non-transitive ‘rock-paper-scissors’ dynamics to be a feature of intragenomic conflict mediated by TA complexes. Intragenomic conflict could be sufficient to select deleterious genes on chromosomes and helps to explain the previously perplexing observation that many TA genes are found on bacterial chromosomes.

## 1. Introduction

Genomes comprise multiple genes that often do not share the same interest [1]. Such genomic conflicts are ubiquitous, and range from conflict over inheritance, such as in the case of genomic imprinting, to conflict over cell division, such as in the case of cancer where cancerous cells reproduce to the detriment of the genome as a whole.

Mobile genetic elements in bacteria provide many interesting examples of intragenomic conflict, characterized by mixed horizontal and vertical routes of transmission. Plasmids are archetypal mobile elements, and can reproduce in tandem with their host (transmitting vertically) but also independently (transmitting horizontally) often at a cost to the host [2]. This mix of transmission routes causes a potential conflict between plasmid persistence and replication, and the interests of the host chromosome [1–6].

**Figure 1.**
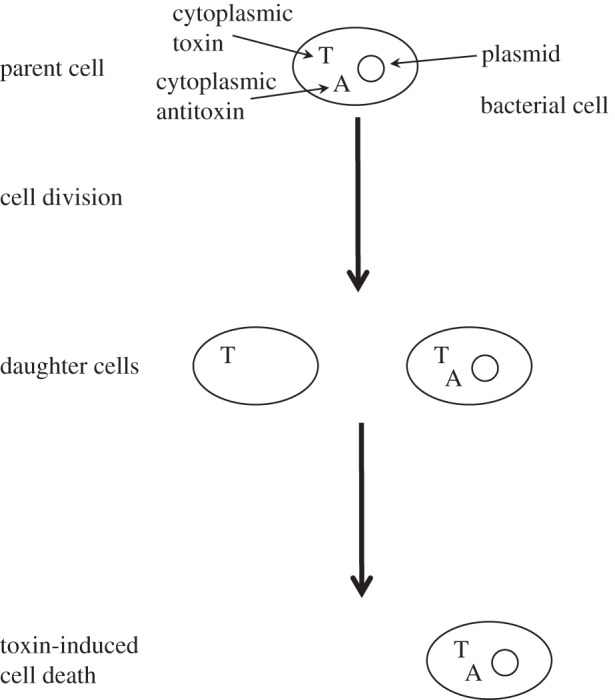
Schematic of plasmid addiction showing the presence (or the absence) of the toxin (T) and antitoxin (A) within the cytoplasm. If the plasmid is lost at cell division, then the antitoxin, which has a shorter half-life, quickly degrades and only the toxin is left. As a result, the daughter cell is killed.

One interesting plasmid-driven conflict is in the case of plasmid-carried toxin–antitoxin (TA) complexes [7–9], where the toxin acts to harm the cell, whereas the antitoxin acts to neutralize the toxin. If the plasmid is lost through segregation at cell division, the stoichiometry of the toxin and antitoxin changes quickly, leading to bacteriostasis or cell death (typically owing to a longer toxin half-life; figure 1). Therefore, carriage of a TA complex does not directly enhance vertical or horizontal transmission, as its principal phenotypic effect (cell death) occurs only following the loss of the complex. As TA systems code for both an antitoxin and a toxin, it is likely that they impose a metabolic cost on the host cell, in the absence of cell death. Despite this, TA systems are frequently found on plasmids [7,8], raising the question of how such a system can have evolved.

A number of studies have shown or proposed that TA systems can be viewed as plasmid persistence adaptations [10–14]. Gerdes *et al.* [7] demonstrated that the loss of TA cassettes induces post-segregational killing (PSK), and argued that TA cassettes therefore function as stability adaptations, ‘addicting’ cell lines to the TA complex [7]. A fundamental concern with the stability/addiction hypothesis is that the PSK phenotype is expressed only following the loss of the replicon. A test of the stability hypothesis showed that TA plasmids are outcompeted by isogenic TA– plasmids (in distinct cell lines) in the absence of conjugation [15]. However, under co-infection (within-host competition), the TA plasmid was able to outcompete and exclude the TA– competitor from a well-mixed population, as now the PSK phenotype fell preferentially on cells carrying the TA– plasmid [15].

Mongold [16] concluded from a theoretical analysis that plasmid-level competition will not select for rare plasmid-encoded TA complexes unless they also carry host-beneficial alleles or have high rates of conjugation, and suggested that plasmid-encoded TAs are coincidental artefacts of gene transfer from chromosomes. Further theoretical analysis by Mochizuki *et al*. [17] illustrated that the rare invasion of TA complexes could, however, be favoured by the spatial structuring of host cells. TA systems also appear frequently on the chromosomes of many bacteria [8,11,18–20]. Different reasons for why chromosomal TA systems would persist have been proposed [11,12], from the role of cell death in biofilm formation [19,20] to their role in bacterial persistence [21,22]. Chromosomally carried TA systems have been shown to confer host resistance to related plasmid-borne TA complexes, as the antitoxin will be present in the cytoplasm even after a TA plasmid is lost through segregation [15,23–26]. TA complexes arise in a variety of different organisms, from genetic drivers in eukaryotes, to addiction complexes in bacterial plasmids, and represent a wider set of genomic conflicts [1]. Here, we build models to describe how plasmid TA complexes evolve, and how they can be maintained in bacterial populations. We show that, given the local establishment of a plasmid-borne antitoxin gene, the full addiction complex can evolve under local competition, but that this remains unstable with respect to host resistance (i.e. chromosomal TA systems) against the toxin. If the TA system is present on both chromosomes and plasmids, this ultimately leads to cycling between TA plasmids, plasmids without the TA complex, chromosomes with the TA complex and wild-type cells.

## 2. Model and results

We build a model to examine the conditions driving the evolution and evolutionary stability of plasmid addiction. We base our model on susceptible and infected (SI) models that have been used extensively to study the evolution and persistence of plasmids and mobile genetic elements [2,4–6,27–29]. We start with a population of plasmid-free hosts, with density being *n*_F_. The density of plasmids that do not carry any toxin or antitoxin genes (which we refer to as ‘null’, or I, plasmids) is denoted *n*_I_ and they are lost from a cell lineage (through miss-segregation at cell division) with a probability *s* and exert a cost *x* (e.g. conjugation), on their host. We assume logistic population growth, where the *per capita* birth and death rate is given by *a* – *μN*. Here, *a* is the *per capita* growth rate, whereas *μ* represents the density-dependent death rate and *N* is the total number of cells in the population. We assume that any costs (such as the cost of bearing a plasmid *x*) are small and manifest themselves as a reduced growth rate. Horizontal transfer of plasmids occurs through conjugation, at rate *β*. The dynamics of wild-type hosts, and hosts infected with null plasmid, are2.1a
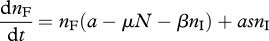
and2.1b
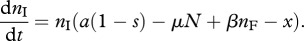


Full details of notation used in the model are given in table 1. Here, *N* is the total population density (i.e. *N* = *n*_F_ + *n*_I_). In the absence of any plasmids, the equilibrium density (carrying capacity) of plasmid-free cells is *n*_F_ = *a*/*μ*. Plasmid-carrying cells will be able to invade this population if *βa*/*μ* > *x* + *as*. Thus, if the rate of horizontal transfer outweighs the costs borne by the plasmid (in terms of the cost of plasmid carriage *x* and the overall rate of segregational loss *as*), a plasmid will be able to invade. This is the general condition for plasmids to persist, which is that the rate of horizontal transfer has to exceed any net costs of harbouring a plasmid [4,6]. More generally, the condition allows for invasion even in the absence of conjugation (i.e. when *β* = 0), if the plasmid carries sufficiently beneficial alleles, such that *x* < 0 and therefore 0 > *as* + *x*. Throughout this paper, we assume that horizontal transfer and/or the carriage of host-beneficial alleles is great enough to favour plasmid persistence, and that therefore *βa*/*μ* > *x* + *as*. This condition does not, however, take into account competition with other plasmids of the same incompatibility group in the population (later in we address this limitation by explicitly considering co-infection). So long as the invasion condition is met, the plasmid-carrying population will increase until the two populations arrive at coexistence equilibrium with plasmid-free cells persisting owing to their continuous generation from carrier cells via segregational loss.

**Table 1.** List of notation used in the model.

symbol	Description
*n*_F_	density of wild-type cells (F)
*n*_I_	density of cells infected with the null plasmid (I)
*n*_A_	density of cells infected with a plasmid bearing the antitoxin gene only (A)
*n*_TA_	density of cells infected with a plasmid bearing the addiction-complex (TA)
*n*_R_	density of cells with the antitoxin on the chromosome (R)
*n*_RAT_	density of cells (RTA)
*a*	*per capita* population growth rate
*μ*	*per capita* density-dependent death rate
*N*	total density of cells in the population (in the full system it is *N* = *n*_F_ + *n*_I_ + *n_A_* + *n*_TA_ + *n*_R_ + *n*_RTA_ + *n*_IT_ + *n*_IR_).
*β*	rate of horizontal gene transfer
*s*	rate of segregational loss
*r*	scale of replacement of killed cell
*x*	cost of bearing a plasmid
*z*	benefit of antitoxin, carried on a plasmid
*c*	cost of plasmid addiction-complex
*y*	cost of antitoxin
*y*_c_	cost of antitoxin when carried on the host chromosome

### (a) Full addiction complex

The plasmid addiction complex kills cells that lose the plasmid through segregation. We assume that cells with the complex pay an additional direct metabolic cost *c*. When the plasmid is lost through segregation (at rate *as*), we assume that the antitoxin is quickly degraded, leaving only the toxin, and therefore the cell dies (so the loss term *as*, representing host death, is qualitatively different from the other costs *x* and *c*, which represent loss of fecundity). We assume that other cells in the population will quickly replace the dead cells resulting from PSK. In contrast, we assume that −*μN* represents losses due to resource limitation and so does not permit immediate replacement.

Following other models, using assortment between strategies to model relatedness [30–33], we introduce the term *r* (where 0 ≤ *r* ≤ 1) to denote the scale of replacement following PSK events. We use such a parameter to keep our model both tractable and general, and we assume that this replacement arises owing to the underlying spatial structure and demography (e.g. motility, life-history characteristics) of the bacteria. The most likely cause of replacement by similar cells will be if there is spatial structure, and thus our parameter *r* can be thought of as describing the level of assortment between strains (as such, our model has similarities to previous models incorporating explicit spatial structure; [17]). If *r* = 1, the dead cell is replaced by a cell carrying the addiction plasmid (‘local’ replacement, e.g. high-spatial structure), whereas if *r* = 0, the dead cell is replaced by a random member of the population (‘global’ replacement, no spatial structure) that is proportional to the frequency of the given cell type in the population (i.e. *n_j_*/*N*, where *j* denotes the strain). To simplify our model, we further assume that cells cannot be co-infected by both null I plasmids and TA plasmids. From these assumptions, the dynamics of cells that contain the addiction complex are therefore2.2a
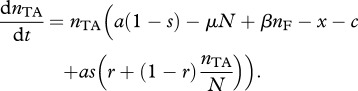


The dynamics of wild-type cells, and cells infected with the null plasmid, are2.2b
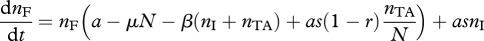
and2.2c
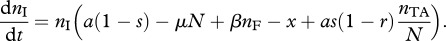


If the wild-type host cells and null plasmids are at the non-trivial (and positive) equilibrium, *n*_F_* and *n*_I_* respectively, the addiction complex will be able to invade if2.3
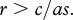


**Figure 2.**
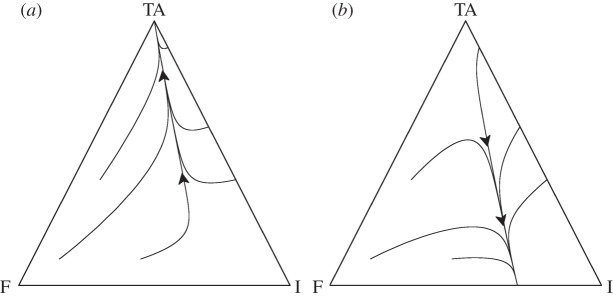
Numerical simulations drawn as phase diagrams in triangular *inset* showing proportions of F, I and TA for (*a*) dominance by a TA encoding plasmid (where *r* = 0.75 and *ars > c*) and (*b*) dominance by a null (non-TA) plasmid (where *r* = 0.1 and *ars < c*). Initial conditions {I(0)*,* TA(0)} = {0.1, 0.1}, {0.1, 0.4}, {0.1, 0.9}, {0.4, 0.1}, {0.4, 0.6}, {0.6, 0.4}. Remaining parameters are *a* = 1 h^−1^, *a/*μ** = carrying capacity = 10^8^ ml^−1^, *β* = 7.5 × 10^−12^ per bacterium h^−1^ ml^−1^, *c* = 10^−4^ h^−1^, *x* = 10^−4^ h^−1^, *s* = 3 × 10^−4^ h^−1^.

As long as this condition holds, the addiction plasmid will both outcompete and be immune to invasion from null plasmids (figure 2). The simple rule, described in inequality (2.3), shows that local replacement is a requirement for the complex to evolve, and high-segregation rates, and low costs of addiction, will favour the evolution of the addiction complex. A critical criterion in assessing inequality (2.3) is the magnitude of segregational loss, *as.* In the absence of co-infection, *as* is due to the rare failure of the segregational machinery during cell division, with estimates of *as* being at least as low as 10^−3^ h^−1^ [34], rendering inequality (2.3) irrelevant for all but the most costless plasmids. In contrast, the rate of segregational loss in co-infected cells is far higher, as the normal functioning of segregational machinery will lead to the rapid separation of incompatible plasmids into distinct lineages, with *s* tending to 0.5 per hour for doubly infected cells [16,34], greatly favouring the likelihood of TA invasion. Later in the study, we explicitly introduce co-infection dynamics.

It is interesting to note the similarity between inequality (2.3) and Hamilton's rule *R* > *C/B* [35]. In this case, *C* is the cost of expressing an altruistic behaviour (in this case, the overall cost *c* of maintaining the addiction complex), *R* is the relatedness (represented by *r*) between an actor (the addiction-carrying plasmid that kills its host after being segregated) and a recipient (a cell which replaces the dead cell), and the benefit of the behaviour *B* of expressing the addiction complex (in this case *as*, the rate of segregation loss of plasmids in the population, dependent in turn on co-infection).

### (b) Separating the complex

The preceding analysis dealt with the TA complex as being a single entity. However, the evolution of TA systems represents a ‘chicken-egg’ paradox: without the antitoxin, the toxin cannot evolve, but the antitoxin has no use in a context lacking the toxin. We therefore now assume that the complex is made up of two separable genes, one coding for the toxin (denoted by subscript *T*), and the other coding for the antitoxin (denoted by subscript *A*). What are the possible evolutionary trajectories of these two genes? To establish a TA complex, we argue that either the T or A gene alone must offer a direct advantage in some but not all of the environments encountered by an evolving plasmid population, thus breaking the deadlock between two individually costly genes. We formalize our argument using the ‘antitoxin-first’ scenario in the following text. In the discussion, we highlight a parallel ‘proto-toxin-first’ solution to this ‘chicken-egg’ paradox.

To build the ‘antitoxin-first’ argument, we begin by assuming that the toxin gene is lethal when alone (we relax this assumption in the discussion). We now assume that *n*_A_ cells are viable, with the plasmid-carried A gene imposing a direct cost *y* and a potential benefit *z*. The dynamics of the antitoxin and the TA complex can then be described by the equations2.4a
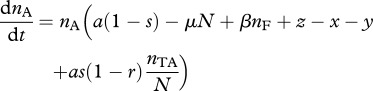
and2.4b
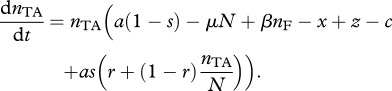


If *z* > *y*, there is a net benefit to the plasmid carrying the antitoxin gene (we separate *y* and *z* in order to explicitly distinguish the costs and benefits of the antitoxin gene). An antitoxin allele could generate resistance or stabilizing benefits *z* to the host–plasmid lineage for a variety of reasons, for example by conferring antibiotic or bacteriocin resistance to the cell.

Modifying equation (2.2*b,c*) to incorporate the additional *n*_A_ lineage, the dynamics of the wild-type cells and null plasmid are then2.5a
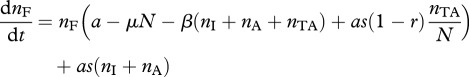
and2.5b
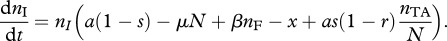


If both wild-type cells, and those infected with the null plasmids, are at the non-trivial (and positive) equilibrium (i.e. *n*_F_* and *n*_I_*), we can see that the antitoxin will invade subpopulations simply if *z > y*. Thus, the antitoxin must generate some direct benefit in order to outcompete the null plasmid. Given a homogenous population of host cells (where *z* is constant across all host cells), and *z* > *y*, the A plasmid will replace null plasmids across all subpopulations, and the resistance or antitoxin gene will be uniformly present across all plasmids. Assuming that the TA plasmids also get a benefit *z* from carrying the A gene on the plasmid (as part of the TA complex), the full TA complex can still invade (when *n*_F_* and n_A_* are at equilibrium) if *ras* > *c*–*y*.

**Figure 3.**
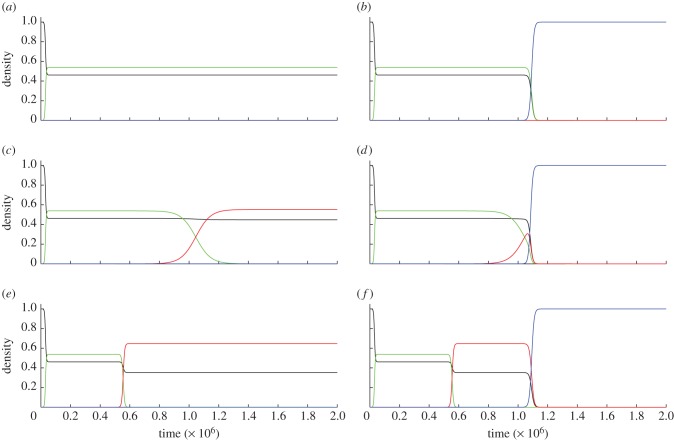
Invasion dynamics of plasmids carrying antitoxin genes (A) and addiction complexes (AT) as a function of antitoxin direct benefit *z* and relatedness *r*. (*a,b*) No direct benefit of antitoxin gene (*z =* 0). (*c,d*) Intermediate benefit (***z*** = 10^−5^). (*e,f*) High benefit (*z* = 2 × 10^−4^). (*a,c,e*) Low relatedness (*r* = 0.25). (*b,d,f*) High relatedness (*r* = 0.75). The population starts off with wild-type cells (F) and null plasmids (I) at equilibrium. After this strains are added from rare: *n*_A_(*t* = 5 × 10^5^) = 10^−5^, *n*_AT_(*t* = 10^6^) = 10^−5^. Other parameters as in figure 2, plus *y* = 0. The line colours and type refer to solid-black: F wild-type cells, solid-green: I null plasmids, solid-red: A antitoxin plasmids, solid-blue: AT plasmids carrying the addiction complex. Densities are scaled to the carrying capacity of uninfected cells (*a/*μ**).

In contrast, if the cellular environments are heterogeneous (and *z* varies across cells in different environments), then the A plasmid can invade subpopulations where A yields a benefit (*z* > *y*, figure 3*c–f*) and fail in others (in particular, where A does not yield a benefit and *z* = 0, and is outcompeted by null plasmids; figure 3*a,b*). The resulting heterogeneous distribution of the A allele provides a context for the emergence and spread of the TA complex. Specifically, the full complex can emerge if plasmids in subpopulations supporting the A plasmid then go on to acquire the toxin gene (figure 3*c*–*f*), and can then expand in subpopulations where *z* = 0 (and thus *n_A_* = 0), whenever *asr* > *c* (figure 3*b*). The cellular heterogeneity results in subpopulations that are locally adapted to some local stressor (e.g. carriage of the A gene in figure 3*e*,*f*). The toxin gene, in combination with the antitoxin, can then invade (figure 3*b*). The importance of environmental heterogeneity and gene-by-environment interactions in breaking ‘chicken-egg’ obstacles to toxin-resistance trait evolution has also been proposed in the context of immuno-manipulative pathogens [36]. In this immunological context, host heterogeneity allows the specialization of pathogen strains on distinct immunological challenges (generating differential resistance or ‘antitoxin’ settings). Strains specialized on the most challenging environments can then invade more benign environments by triggering the environmental stressors to which they are already adapted (via immuno-manipulation or, in the present study, toxin production). TA loci may evolve in an analogous manner. A diverse array of plasmids are known to code for specific resistance phenotypes to heterologously distributed environmental stresses [2,37,38]. In consequence, these resistance determinants (i.e. A plasmids) are locally adaptive in a restricted and challenging subset of environments. Plasmids that encode resistance determinants in response to a locally present toxin may then co-opt expression of the toxin, permitting the TA encoding plasmid to invade benign environments lacking resistance whenever *ars > c*.

### (c) Host resistance to the toxin

Following the invasion and dominance of a host population by a TA plasmid, we now ask whether a host resistance trait (i.e. a chromosomally coded antitoxin) can evolve in benign environments lacking the exogenous stressor (i.e. *z =* 0; if the direct benefits of the A gene are sufficiently large, then there is little puzzle surrounding its acquisition, even in the absence of circulating TA plasmids). One simple mechanistic route to the establishment of a chromosomal antitoxin gene is via transposition from plasmid to the chromosome of A and/or TA. TA systems are frequently observed to be carried on bacterial chromosomes [8]. Host bacteria carrying the TA system chromosomally can potentially resist the lethal effects of TA plasmid loss, as the cell retains the ability to produce the antitoxin [15,23–26].

To begin our analysis of host resistance, we consider the case where the TA plasmid has gone to fixation (see equation (2.2*a*)), at an equilibrium density *n*_TA_* = (*a* – *c* – *x*)/*a*. We now consider a rare population of resistant-uninfected hosts *n*_R_, which upon infection with the TA plasmid will generate a lineage of infected resistant hosts *n*_RTA_. Note that ‘resistance’ implies only that the host lineage can survive the loss of the T-encoding plasmid, and does not imply any resistance to infection. The density of individuals with resistance on the chromosome is given by2.6a
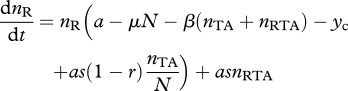
and2.6b
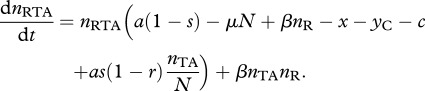
Here, *y*_c_ is the cost of carrying the resistance determinant on the chromosome. We assume that the cost of the antitoxin alone is less than the direct costs of the full TA. We can look at the invasion criteria by considering the Jacobian matrix of equations (2.6*a*) and (2.6*b*). The resistance gene will invade if the dominant eigenvalue of this matrix is positive, evaluated when *n*_TA_ is at equilibrium, *n*_TA_* = (*a* – *c* – *x*)/*μ*, and *n*_R_ → 0 and *n*_RTA_ → 0. This is the case if:2.7
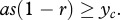


Inequality (2.7) suggests that lower spatial structure (i.e. lower *r*) and higher segregational loss (higher *as*) favours the evolution of host resistance. Interestingly, if *as* – *y_c_* > *asr* > *c*, we predict cycling among strains. Specifically, a plasmid carrying the TA complex will be able to invade a population of null plasmids, after which any cell that develops resistance to the TA plasmid will also be able to invade. Once resistance establishes itself in a population, a null plasmid (or a plasmid only with the antitoxin gene) will be able to outcompete a TA plasmid as null plasmids do not pay the cost *c* of expressing the TA complex (as long as *c* > 0). Once null plasmids have invaded, wild-type cells will be able to invade, outcompeting cells with TA on the chromosome (as they do not pay the cost of the complex on the chromosome, *y*_c_).

### (d) Numerical simulation of full dynamics

The interactions between strains *n*_F_, *n*_TA_ and *n*_R_ display a non-transitive form of competitive advantage labelled ‘rock-paper-scissors’ dynamics after the popular children's game [39]. In our case, *n*_TA_ beats *n*_F_ and *n*_I_, *n*_R_ beats *n*_TA_, *n*_F_ beats *n*_R_, etc. These non-transitive interactions are closely akin to three-strain models of bacteriocin production in bacteria, where killer (TA), resistant (antitoxin only) and sensitive (neither) can cycle and potentially coexist in spatially structured populations [39–42]. This highlights the non-transitivity of TA systems in general, and strain cycling will be common regardless of whether the TA complex is involved in the production of bacteriocins or in PSK. To examine whether TA systems could lead to cell cycling, we built (using Matlab) a numerical model with the dynamics described earlier.

**Figure 4.**
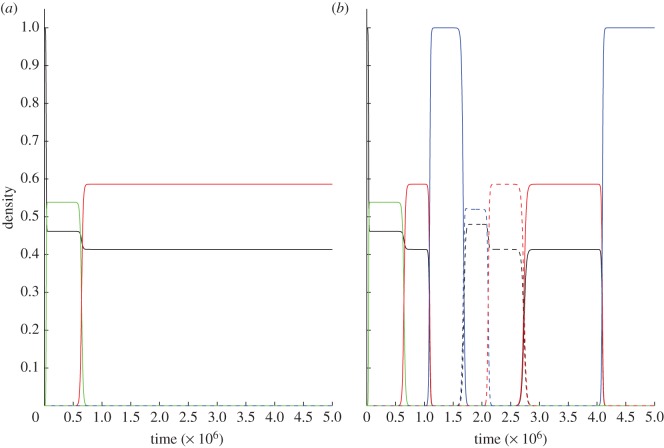
Full dynamics of the system illustrating cycling between strains for (*a*) the case where addition cannot invade (‘low’ relatedness, *r* = 0.25) and (*b*) for the case where plasmid addiction can invade (‘high’ relatedness, *r* = 0.75), and drives strain cycling. The population starts off with wild-type cells (F) and null plasmids (I) at equilibrium. After this strains are added from rare *n*_A_(*t* = 5 × 10^5^) = 10^−5^, *n*_AT_(*t* = 10^6^) = 10^−5^, *n*_R_(*t* = 1.5 × 10^6^) = 10^−5^, *n*_RA_(*t* = 2 × 10^6^) = 10^−5^, *n*_F_(*t* = 2.5 × 10^6^) = 10^−5^, *n*_I_(*t* = 3 × 10^6^) = 10^−5^ *n*_AT_(*t* = 3.5 × 10^6^) = 10^−5^ and *n*_AT_(*t* = 4 × 10^6^) = 10^−5^. Other parameters as for figure 2, plus *z* = 7.5 × 10^−5^, *y* = 5 × 10^−5^, *y*_c_ = 0. The line colours and type refer to solid-black: F wild-type cells, solid-green: *I* null plasmids, solid-red: *A* antitoxin plasmids, solid-blue: +*AT* plasmids carrying the addiction complex, dotted-black: *R* plasmid-free cells with resistance on the chromosome, dotted-red: *RA* cells with resistance on the chromosome carrying an antitoxin plasmid, dotted-blue: *RAT* cells with resistance on the chromosome carrying the addiction plasmid.

The results of the initial invasion of the addiction complex can be seen in figure 3*b*. This shows that a population of plasmid-free wild-type cells is first invaded by a null plasmid, and then is invaded by the TA complex that then dominates the population and excludes all other cells. Figure 4 shows the dynamics of our full model, where the antitoxin can be carried on the chromosome, for both low relatedness (figure 4*a*, *r* = 0.25) and high relatedness (figure 4*b*, *r* = 0.75). In the case of low relatedness, wild-type cells and cells carrying the antitoxin plasmid prevail, and cannot be invaded by other types (figure 4*a*), while when relatedness is high (and inequality (2.3) is fulfilled), the TA plasmid can invade (as shown in figure 4*b*).

**Figure 5.**
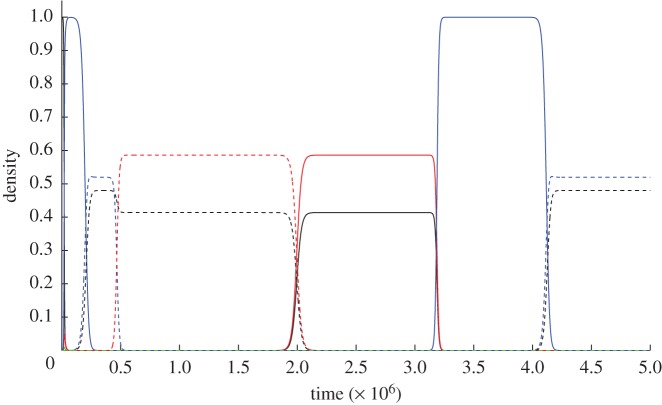
Numerical simulation of the full model described in appendix S1, illustrating non-transitive dynamics. Wild-type cells start at equilibrium density (*n*_F_ = 1) and all other cells begin at a low density in the model (i.e. 10^−4^). Solid grey lines represent plasmid-free cells with resistance on the chromosome while dotted grey lines represent cells infected with plasmids carrying the addiction complex. Parameters as for figure 4. The line colours and type refer to: solid-black: *F* wild-type cells, solid-green: *I* null plasmids, solid-red: *A* antitoxin plasmids, solid-blue: *AT* plasmids carrying the addiction complex, dotted-black: *R* plasmid-free cells with resistance on the chromosome, dotted-red: *RA* cells with resistance on the chromosome carrying an antitoxin plasmid, dotted-blue: *RAT* cells with resistance on the chromosome carrying the addiction plasmid.

However, in this case, once the addiction plasmid has invaded, a cell with the antitoxin encoded on the chromosome can invade. The resulting population also harbours the addiction plasmid, but at a lower density, and in combination with plasmid-free cells carrying the resistance gene. After this, a plasmid with the antitoxin gene invades, which facilitates the invasion of plasmid-free wild-type cells, coexisting with plasmids carrying the antitoxin gene. Once this state of coexistence is reached, the addiction complex can invade once again. Both our analytical models, and the numerical simulation in figure 4*b*, demonstrate that plasmid addiction will involve cycling between the different genes and that, over longer evolutionary time-scales, addiction complexes are inherently unstable. The numerical results presented in figures 3 and 4 introduce rare strains at regular intervals (every 10^4^ generations) in order to demonstrate that cycling is possible between strains. To test the robustness of cycling to random introduction of new strains, we modified our model to start with all strains present at a low density (i.e. with a density of 10^−4^). Interestingly, null plasmids never invade in this model, as they are always outcompeted by A plasmids. Figure 5 shows that addiction complexes, when carried on the chromosome and on a plasmid, can give rise to strain cycling.

### (e) Co-infection and segregational partitioning

We earlier demonstrated that the ability of a TA plasmid to invade a resident null plasmid population would depend critically on the rate of segregational loss (inequality (2.3)), and we commented that this rate will in turn depend on the prevalence of co-infection. While our inequality (2.3) can be interpreted generally (with *s* varying with the extent of co-infection), we now explicitly explore the synergistic interaction between within-host competition and spatial structuring.

Here we assume equal partitioning, wherein cells infected with two different plasmids give rise to one daughter cell with one plasmid and one daughter cell with the other plasmid. As shown earlier, we assume that if the TA plasmid is lost from a cell, as is the case when the daughter cell inherits only the null plasmid from a cell containing both the TA and the null plasmid, then that daughter cell will be killed. We further retain the previous assumption that, if a cell is killed by PSK, another viable neighbouring cell (I or TA) will replace the dead cell based on the genetic structure *r* of TA-carrying cells. In addition, we assume no miss-segregation and no wild-type strains (i.e. *s = n*_F_ *=* 0). As all bacteria now carry plasmids, we simplify the notation by describing the maximal growth rate as *α* = *a* − *x*. Together, these assumptions yield the following dynamical equations2.8a
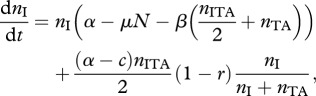
2.8b
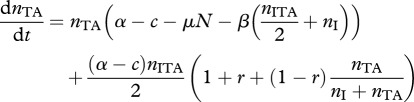
2.8c
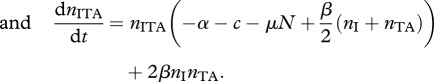


In order to evaluate whether the TA system will be able to invade from rare, we take the Jacobian matrix of equations (2.8*a*–*c*), evaluated at {*n*_I_ = *α*/*μ*, *n*_TA_ *= n*_ITA_ *=* 0}. If the dominant eigenvalue of this matrix is positive, then a rare TA system will be able to invade, which is the case when the following inequality holds:2.9
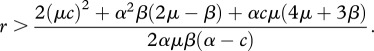


In qualitative agreement with our earlier inequality (2.3), we see that the TA complex can spread if relatedness is sufficiently high (inequality (2.9)). More generally, a little algebra shows that this critical value r* is monotonically decreasing with *β*, and when *β* equals zero, the TA complex cannot invade for any cost. This result implies that increasing transmission favours the kin-selected advantage to an invading TA lineage, owing to the increasing incidence of co-infected cells (higher *s*, in the terms of inequality (2.3)).

## 3. Discussion

Intrageneomic conflict can be found in all genomes [1]. Our model examines the evolution of toxin and antitoxin systems in plasmids, and the role they may play in intragenomic conflict. Our results demonstrate that a key way that these costs can be mitigated is if, once a cell dies as a result of the complex, it is more likely to be replaced by a cell carrying the same complex. This is also true of the model incorporating partitioning between incompatible plasmids: relatedness is a key component of whether TA systems can evolve. TA plasmids can invade if there is a sufficiently high chance, *r*, that the dead cell will be replaced by a cell carrying the TA complex. If a cell is killed by the TA complex and replaced at random from the entire population, then the cost of bearing the TA complex cannot be outweighed by the gain brought by killing cells not carrying the plasmid. Genetic structure of the plasmid population (generated both by structuring of the bacterial population and by co-infection of neighbouring bacteria) is therefore an important component of plasmid competition and the evolution of TA complexes. The *r* in our model is equivalent to relatedness (and can be seen as a measure of assortment between strains, or genetic similarity between the cell killed by PSK and the strain which replaces it; [32,33]). In the case of plasmids, not only does spatial structure increase associations between local cells, but the act of transmitting plasmids can also increase such genetic structure [43,44].

Understanding how TA complexes arise has been a perplexing issue so long as the antitoxin serves no benefit in the absence of the toxin, and the toxin only acts to harm the cell (and the plasmid itself). No matter the benefits of the combination of toxin and antitoxin, if each trait alone is costly, then the stepwise evolution of the combined complex remains problematic. We argue that this apparent barrier to a stepwise evolution of TA complexes can be overcome if one of the traits offers a direct advantage in some but not all the environments encountered by an evolving plasmid population [36]. In the results section, we outline an ‘antitoxin-first’ scenario, where the A gene serves some environmental resistance function that is beneficial in some stressful subset of host environments (figure 3*c*,*d*). Consistent with this scenario, plasmids often confer resistance to patchily distributed environmental stressors [37,38]. From this widespread plasmid/A (resistance) association, we conjecture that plasmid/TA associations are an elaboration involving the recruitment of a T gene to extend the local advantage of the partner A gene into new, less extrinsically stressful environments, that cannot support the antitoxin trait alone (figure 3*b*).

Thus far, we have assumed the toxin is harmful in all contexts. Now, we relax this assumption in order to consider a ‘toxin-first’ route to TA complex evolution. The apparent toxicity of certain genes can be context-specific, where the gene product may confer a lethal, deleterious or even beneficial phenotype depending on the environment. For example, conditionally expressed proteins that transport nitrogenous compounds when preferred sources of nitrogen are limiting can be both adaptive and either deleterious or lethal depending on the environment. In a nitrogen-limiting environment in which amino acids may be prevalent, an amino acid transporter is adaptive. However, in the same environment with toxic amino acid analogues, expression of the transporter may be deleterious or lethal [45]. Similarly, transporters can suppress the effects of mutations in biosynthetic pathways, being adaptive when the essential metabolite cannot be made, but deleterious when a toxic analogue is also present [46]. In both cases, toxicity is an environment-dependent side-effect of an otherwise adaptive trait. Similar to the former case of an initially adaptive antitoxin, environmental heterogeneity (in this case, heterogeneity in toxicity) permits the gene that can have a toxic effect to exist in a subset of environments without the need for a coupled antitoxin. Therefore, in the absence of an antitoxin, the toxin gene is constrained to environments where it is benign. However, when the toxin and antitoxin genes are linked on a plasmid, the plasmid is able to invade populations of cells that lack resistance to the toxin whenever *ars > c*. The ‘toxin-first’ scenario is of potentially greater importance for the evolution of type 1 TA systems where the antitoxin (antisense RNA) presumably has no function besides suppressing toxin expression. In both evolutionary scenarios, environmental heterogeneity (of either T or A effect) is necessary to explain the evolutionary origins of TA systems on plasmids. Once TA plasmids evolve, they can potentially have beneficial effects on bacterial host populations. For example, plasmid-carried TA complexes have been found to be associated with microbial public good production (secreted proteins), and can be viewed as a mechanism to enforce costly cooperative behaviours [27,43,47,48].

Following the spread of a functional TA complex in a host population owing to sufficient local replacement of killed cells (i.e. *asr* > *c*), the establishment of an antitoxin gene on the host chromosome can be favoured, which protects cells in the event of segregational loss of the TA plasmid [15,23–26]. If this antitoxin gene is otherwise costly to the host cell, selection on this gene is likely to be reversed as soon as the TA plasmid is sent sufficiently into decline, in turn favouring the subsequent invasion by the susceptible wild-type and thus opening the potential for ‘rock-paper-scissors’ cycling coexistence of sensitive, killer and resistant strain-types (figure 3) [39,42]. This can help us to explain the widespread occurrence of TA genes on bacterial chromosomes [8,12]: if a TA complex jumps from the plasmid to a chromosome, that host will have the potential to reduce the effects of the antitoxin, and thus avoid PSK. While many alternative explanations have been given for the occurrence of chromosomal TA systems [12], our model is the first to examine their role as a coevolutionary mechanism to avoid PSK from TA plasmids and is in accordance with empirical studies that show chromosomally encoded TA loci prevent within-host competition [24]. These results are also eminently testable via simple competition experiments, as the predicted non-transitive competitive hierarchies translate directly into invasion experiments: rare *n*_TA_ can invade resident *n*_F_ and resident *n*_I_; rare *n*_R_ can invade resident *n*_TA_; rare *n*_F_ can invade resident *n*_R_.

From the perspective of a given plasmid, segregational loss and cell death are equivalent (because both result in an equivalent loss of direct fitness to the plasmid). As our model shows, plasmids gain an indirect benefit from harming their host when other isogenic plasmid-carrying cells benefit: while PSK has immediately negative consequences for its ex-host, it confers an indirect benefit on other isogenic plasmids by increasing their local frequency. It is worth noting that the similarity between TA systems on plasmids and cytoplasmic incompatibility (CI). For example, in the bacteria-induced CI found in many insects, bacteria such as *Wolbachia* are transmitted only through the female line, i.e. through eggs rather than sperm [49]. This has led to adaptations in bacteria to kill males produced by a female, in order to favour their spread to the next generation. It is widely acknowledged that spatial structure plays an important role in the evolution of CI [50–53]. Bacteria that induce CI do not benefit directly from killing males, as they are not transmitted through the male line. In a similar way, TA plasmids do not gain an advantage from killing cells that no longer carry a plasmid. In both plasmid addiction and CI, it is necessary that related individuals (either CI-carrying females or TA-carrying cells, respectively) benefit from PSK.

Our model goes beyond the scope of other models [15–17] by examining both the origin and persistence of the full TA complex from its antitoxin and toxin-components, and exploring the notion that the widely observed prevalence of TA systems on bacterial chromosomes [8] could be a host adaptation to plasmid addiction complexes. Coevolutionary arms races are common in many systems [54–57], and it is likely that hosts will try to resist the costs of PSK inflicted by TA complexes.
